# Causal Inference in the Age of Decision Medicine

**DOI:** 10.4172/2153-0602.1000163

**Published:** 2014-10-09

**Authors:** A Yazdani, E Boerwinkle

**Affiliations:** Human Genetics Center, University of Texas Health Science Center at Houston, Houston, TX, USA

**Keywords:** Structural equation modeling, Coronary heart disease, Assignment mechanism

## Abstract

Causal analyses and causal inference is a growing area of biostatics. In parallel, there is increasing focus on using genomic information to guide medical practice, i.e. personalized medicine or decision medicine. This perspective discusses causal inference in the context of personalized or decision medicine, including the assumptions and the concept that the task is different depending on whether the primary goal is the average response of treatment in the population or the ability to characterize the response for an individual or a subgroup. This perspective provides a tutorial of modern causal inference and then provides suggestions how application of specific kinds of causal inference would promote advances in translational sciences. The concept of the subpopulation causal effect is one path toward improved decision medicine. A dataset containing cardiovascular disease risk factor levels and genomic information is analyzed and different causal effects are estimated.

## Introduction

Students are taught the perils of inferring causality from observational studies, and the shortcomings of nonrandomized clinical trials. In his seminal text book on modern epidemiology, Rothman et al. [1] dedicate considerable discussion to causal inference and even goes so far as to try to present and critique criteria necessary to consider or establish when concluding a causal relationship between two variables. Although formal conceptualization of causal inference began early in the last century, there remains disagreement concerning the ability to discovery novel causal effects from all but the most rigorous of controlled clinical trials and mechanistic experiments. Causality is connected to probability by some experts (e.g. [2], and [3]), whereby an attempt is made to quantitate the probability that A causes B, with assumptions about the mechanism by which individuals were assigned to levels of A. If that probability exceeds some threshold, a causal relationship is claimed. However, interpreting probabilities as causal quantities in the absence of clear knowledge about the assumptions underlying, this interpretation can lead to confusion. To avoid such confusion, Pearl promoted a deterministic interpretation of causal inference at the population level using structural equation modeling [4–6]. Rubin also defines the causal effect deterministically, but at the individual level [7]; for discussions on causal effect definition see also [8–11] and for causality in genetics effects see [12–13].

The difference between the “individual” and “population” causal effect has meaning that transcends esoteric or theoretical considerations. As an example, let’s consider a drug, a desired outcome and an adverse event. The policy arm of health care wants to know whether prescribing the drug to the population of patients will increase the frequency of the desired outcome (and presumably reduce disease incidence) without undo increase in the frequency of the adverse event. The physician, on the other hand, wants to know whether prescribing the drug to the patient in his/her office at that time will elicit the desired outcome without leading to the adverse event in that patient. Typically, analyses and inference are done on a large sample from the population and then the results are used to make inference about whether the next individual sampled from the same population will respond or not. In its simplest form, inference about the response of the next individual sampled from the population is the average response in the population. Personalized medicine connotes the idea that treatment has been tailored to specific characteristics of the individual. In practice, treatment is not tailored to each individual, but rather is tailored to groups of individuals based on the results of specific diagnostic information, such as the level of a biomarker or genetic information. The term “Decision Medicine” has recently been suggested, which indicates a more immediate translational perspective [14].

The question we ask is whether we should approach causal inference including the assumptions and data analysis task differently depending on whether our primary interest is the average response of treatment in the population or the ability to characterize the response for an individual or a subgroup. Regardless of the term, the field of personalized medicine has much to benefit from advances in causal inference. This perspective provides a tutorial of modern causal inference and then provides suggestions how application of specific kinds of causal inference would promote advances in translational applications of personalized or decision medicine. The example application is carried out pragmatically using a graphical approach followed by Structural Equation Modeling (SEM).

### A short tutorial on causal inference

Because causal inference is a challenging concept for many clinicians and researchers, even those trained in biostatistics, it is informative to reconsider the relationships among prediction, causation and association. Knowing the menu of causes of an outcome improves prediction above what may be done with a set of variables that are only associated with the response variable. Clearly, causation facilitates prediction, but the ability to predict does not imply causation because of the ubiquitous presence of association. The general dogma is that causal variables are better predictors of response compared to variables that are associated with response only because of their correlation with the causative variables. In some cases, these relationships have been mathematically formalized, [15]. Contrary to this dogma, a single associated variable may be a better predictor of average response in the population than a single causal variable because the associated variable may be correlated with multiple causal variables. In addition, a good predictor of population relationships may not be a good predictor of the response to an intervention. A causal variable, on the other hand, can be both a good predictor of population relationships and a good predictor of the response to an intervention.

The relationship among prediction, causation and association is more than just intellectual curiosity when we consider the results of an intervention or randomization. A classic example is offered by HDL-cholesterol, triglycerides and Coronary Heart Disease (CHD). HDL-cholesterol and triglycerides are negatively correlated. For many years, cardiovascular scientists believed that HDL-cholesterol was causally protective of CHD because of reverse cholesterol transport. It even had the nickname “good cholesterol”. However, recent intervention studies with drugs that raise HDL-cholesterol show that CHD rates were not changed by the intervention [16]. Similarly, Mendelian randomization studies with genetic variants that raise HDL cholesterol levels do not affect CHD risk [17]. These data have caused a shift in thinking that the causal variable is triglyceride levels, not HDL-cholesterol; HDL-cholesterol was only associated with the disease through its inverse correlation with triglycerides.

In clinical or human population studies, a causal effect of a treatment or exposure is defined as the difference between the proportion of individuals in the population with the outcome of interest where all individuals have been exposed or treated, *E*(*Y* (*t*)), and the proportion of the same individuals with the outcome of interest where all individuals have not been exposed or treated, *E*(*Y*(*c*)) [18]. As one can see from this definition, we must have 2N measurements in a population of N individual, which is problematic and has been called “fundamental problem” [19]. In each experiment, we can at most observe N responses on n_1_ treated and n_2_ untreated individuals (N=n_1_+n_2_), such as that in a classic placebo-controlled clinical trial. A comparison of responses between the treated and untreated groups must be done on individuals that are the same for all relevant variables, and the degree to which they are not the same dictates the need for covariates and an understanding of confounding. Alternatively, we can observe 2N responses in N individuals treated serially, such as that in a crossover study.

A key element in elucidating the causal effect is understanding bias in the Assignment Mechanism (AM). The AM is the system or structure used to assign treatment to individuals, which is formalized by Rubin [7]. See the accompanying text box for definitions of other words related to causal inference. To discover the causal effect between treatment and outcome, we need to understand the assignment mechanism (i.e. data generating process) as completely as possible. An experimental study is one in which the assignment mechanism is known, such as a randomization clinical trial. Randomization is a tool to eliminate confounding and, therefore, randomization is a favorite study design of clinicians and population scientists desiring to make causal inference. Since the assignment is done randomly, the covariates should be balanced between treated and untreated groups and any observed difference in the outcome is ascribed to the effect of treatment. With randomization, a comparison of the conditional probabilities *P*(*Y*=*y*|*T*=*t*) and *P*(*Y*=*y*|*T*=*c*), leads to causal inference about treatment T on outcome Y. However, the main reason for the ability to make causal inference based on this comparison is an understanding of the assignment mechanism (i.e. randomization), not an understanding of the conditional probabilities. Lack of complete understanding of the assignment mechanism of treatments to individuals in observational studies is one reason that causal inference is difficult in common epidemiologic settings. The interested reader is encouraged to investigate the contrasting conclusions from observational studies and controlled clinical trials for hormone replacement therapy and coronary heart disease in women [20]. Although randomization leads to causal inference, it is often not practical or even desirable in the case where there may be an obvious alternate optimum treatment assignment [21]. So, there is a need to pursue methods and criteria for causal inference in observational and other study designs.

#### Definitions of Common Terms in Causality

##### Causal effect

To define the causal effect, we need to first indicate what we intend to do with the information, and possible responses include:

1. We seek the effect of treatment over the population; this effect is considered as a **property** of the treatment.
2. We are interested in individual causal effect which is seen as the individual **reaction** to the treatment.
3. We are interested in subpopulation (treatment-variable) causal effect which is a result of an **interaction** between the subpopulation stratifying variable and treatment.

##### Covariates

Variables influential to individual’s response other than the primary variables of interest are called covariates; covariates are causes of response. The level of a covariate is to be measured or inferred for each individual

**Treatment assignment mechanism** is a system or process to assign treatment to individuals and we need to clarify this system in order to discover the causal relationship between treatment and response.

##### Illumination assumption

A complete understanding of how individuals have been assigned to treatment or how observations (data) have been generated.

##### Confounders of assignment mechanism

Confounders are covariates that influence both the response and the level of treatment. As a result, the distribution of confounders is different in treated and untreated groups.

### The status quo: the population causal effect

Usual practice, with just a few exceptions, considers all individuals to be the same and differences in response among individuals to be dealt with by increasing dose or identifying alternative or add-on treatments. Classic examples of this approach exist within the majority of professional guidelines (e.g. American Heart Association cholesterol and blood pressure treatment guidelines) that are ubiquitous and impactful, but rarely take into account individual patient characteristics. In this case, we measure the causal effect over the population. To estimate the population causal effect, the response variable measured in a sample of treated and untreated individuals are compared. This is interpreted as causal effect if other covariates are balanced or randomized between the treated and untreated groups. A major source of confounding is the AM - the way in which each individual has been assigned a treatment level. In a randomized clinical trial, treatment is assigned to an individual according to a specified process, and, therefore, the AM is known. In observational studies, there are questions as to why some individuals have been provided a specific level of treatment and some have not, and confounding by indication is a threat to rigorous inference [22]. Before proceeding to the more complicated cases of individual and subpopulation effects, it is instructive to present the population causal effect more formally.

Assume we have N individuals in the population under study. If all N individuals are assigned to the treatment group, we observe *Y*(*t*) = (*Y*_1_(*t*), …, *Y_i_*, …, *Y_N_*(*t*)), and if all N individuals are assigned to the control group, we observe *Y*(*c*) = (*Y*_1_(*c*), …, *Y_i_*(*c*), …, *Y_N_*(*c*)). The quantity *E*(*Y*(*t*)−*Y*(*c*)) is introduced as the population causal effect, where *Y*(*t*) and *Y*(*c*) are potential responses of the entire population under treatment and control, respectively [23]. Since the reactions of all N units under both levels of treatment are considered, the quantity leads us to the population causal effect. However, in most applications, treatment and control cannot be assigned to each individual; and we observe some values of vector *Y*(*t*) and some values of vector *Y*(*c*) regarding the actual treatment assignment, and not the two components for each individual. For the quantity *E*(*Y*(*t*)−*Y*(*c*)) to be interpreted as a valid population causal effect, there are two categories of assumptions that must be considered: monitoring and illumination [24]. The monitoring assumption is satisfied if there is no interference either from the study subjects or from external factors. Violating the monitoring assumption means that the difference between treatment and control responses cannot be solely ascribed to the treatment. In statistics, the monitoring assumption is called Stable Unit Treatment Value Assumption or SUTVA [25]. Clarification of the AM (i.e. illumination) represents a complete understanding of how individuals have been assigned to treatment or how observations (data) have been generated, which may be formalized by potential outcomes called ignorability or conditional ignorability. Ignorability means treatment is assigned independently of an individual’s reactions, *T* ⊥ (*Y*(*t*), *Y*(*c*)) [22].

Let variable *Y_obs_*(*t*) stand for the observed components of *Y*(*t*) and variable *Y_obs_*(*c*) stand for the observed components of *Y*(*c*). The notation *AM* (*K_R_*) is introduced as the causal element [24]. *K_R_* comprises any knowledge related to response. Through a better understanding of *AM* (*K_R_*), we can identify a realization in which the responses are proper for causal inference. In the population in which the causal element *AM* (*K_R_*) has been identified and the treated and control units have been observed, we are able to infer causal effects by comparing the following two quantities,

$$P(Y_{\mathrm{obs}}(t)=y|AM(K_{R})=f)\&P(Y_{\mathrm{obs}}(c)=y|AM(K_{R})=f).$$

Both quantities are conditioned on *AM (K_R_)=f* which means the assignment mechanism has been identified regarding knowledge related to response, and the assignment mechanism is the same over the population. It is important to note the causal element *AM* (*K_R_*) cannot be replaced by a design variable or an adjusting covariate. The notation *AM* (*K_R_*) means the assignment mechanism has been fully identified by considering knowledge related to the response, thus conveying more information than conditioning on a covariate.

The assignment mechanism can be illustrated by causal graphs and the causal effect can be estimated by structural equation modeling which is compatible with causal graphs [4]. We use this framework in the example application provided at the end, because it is practical in biomedical and translational settings.

### Personalized medicine and the individual causal effect

Personalized medicine is the ability to use an individual’s genetic make-up and life experiences to better maintain health, diagnose and treat disease, and avoid adverse outcomes resulting from treatment. Similarly, we interpret the individual causal effect of a treatment as the expected response of each individual to the treatment in comparison with its response to the control. The individual causal effect differs from the population causal effect because the expected response is no longer the same among individuals. To formalize the individual causal effect, *Y_i_*(*t*) and *Y_i_*(*c*) are defined as the response of individual *i* to the treatment and control, respectively. A comparison of the potential outcomes of individual *i*, such as *Y_i_ (t) - Y_i_(c)*, gives us the causal effect of the treatment on individual *i*. In most cases, however, only one of the potential outcomes is observed and the other is missing. To avoid this fundamental problem, we often consider one unit over time and assume that the effects of relevant covariates are held constant, such as in a typical cross-over design. The causal effect *Y*(*t*) – *Y*(*c*) is a random variable whose value is different for each individual and has variance σ^2^_t_ in the population.

In the contemporary era of genomics, personalized medicine has become synonymous with genomic medicine, and the individualized effects of treatment are thought to be due to genetic differences among individuals. Considering an individual’s genotype as a random effect influencing the level of the phenotype and creating correlations among related individuals is as old as the field of human genetics itself [26]. However, it has only been recently that the technology exists to begin to estimate the level of an effect, such as, for an individual. Genome-wide association studies using dense genotyping arrays (e.g. [27]), exome sequencing [28], or whole-genome sequencing [29] are beginning to identify the genes and alleles influencing many medically-relevant traits, including treatment response. Early attempts to model *Y_i_*(*t*) considered only a few loci and created a genetic risk score for each individual [30]. More recently, Visscher and et al used genetic markers spanning the human genome to estimate the heritability of a trait, such as treatment response [31]. To the extent that *Y_i_ (t)-Y_i_ (c)* is genetic, the heritability of a trait is a function of σ^2^_t_. However, these methods do not allow for accurate estimation of *Y_i_*(*t*) for an individual.

If the response to treatment is a unique characteristic of the individual that cannot be predicted *a priori*, then true personalized medicine has little practical utility in medicine or biomedical research. Because every individual is unique, it is difficult to achieve evidence-based personalized medicine. In addition, individualized treatment strategies would be cost-prohibitive. Instead, personalized medicine is being replaced by decision medicine in the practical setting. Decision medicine is different than traditional medicine, which typically operates under the assumption of a population causal effect, and personalized medicine, which considers an individual causal effect. Decision medicine, on the other hand, uses available information to categorize individuals into groups based on the results of formal or assumed interaction analyses, and then considers the population causal effect within a group. Such interaction causal effects are discussed in the next section.

### Decision medicine and the subpopulation causal effect

Between the two extremes of considering the effect of treatment to be the same for each individual (i.e. population causal effect) and the effect to be a unique characteristic of each individual (i.e. individual causal effect) lays the practical domain of the subpopulation causal effect. The subpopulation causal effect simply means that the causal effect of treatment on groups (i.e. subpopulations), within the population, are different for each group. The subpopulation causal effect is an underlying principal of pharmacogenetics and decision medicine, where the groups may be defined by genotype or other relevant characteristics (e.g. gender). The groups may be defined based on the results of a statistical test of interaction between the treatment and a covariate or based on *a priori* biologic or clinical knowledge. The subpopulation causal effect is a practical compromise between the population and individual causal effect, which either do not take into account the unique characteristic of each individual or do show a practical path forward, respectively.

To discover an interaction effect, the illumination of the AM is not enough because there might be an interaction between *T* and a covariate *Z* on response, while Z does not vary with treatment. In such a case, we need to consider *Z* as an effect modifier. However, the illumination of the AM does not provide this information because Z is not a confounder of the treatment AM. All of this leads to fundamental challenges for the applied practitioner of causal inference; for each covariate we must consider the possibility that it is modifying the effect of treatment (i.e. interaction) [10].

To make the difference between the population and subpopulation effects more tangible, we consider a simple linear model. To find the population causal effect of T on Y, only the potential confounders of the treatment AM denoted by X are modeled, *y* = *α* + *βt* + *γx* + *u*. In this equation, *u* is a realization of *U* which includes variables independent of *T,* and stands for population causal effect of treatment on response *Y*. Now consider that there might be an interaction between *T* and a covariate *P* on response *Y: y* = *α* + *β′t* + *γx* + *ηp* + *λt.p* + *u′* where *U′* is independent of *T*. The subpopulation causal effect is a combination of the effect of T alone and the effect of an interaction between T and a covariate, *P*. In the equation below, we see the difference between the coefficients of *T* in the two above equations:

$$\beta =\beta^{\prime }+\lambda \cdot P(p=1).$$

The effect of treatment in the class of *P*=1 is *β′* + *λ* and the effect of treatment in the class of *P*=0 is *β′*. If *λ* is positive and we do not classify individuals regarding P, we will overestimate the effect of treatment for individuals in the group *P*=0 and underestimate it for individuals in the group *P*=1.

### Example application

In practice and in real data applications, the concept of causal inference is best visualized as a Bayesian Network. In addition, the Bayesian network framework facilitates both estimation and hypothesis testing (i.e. statistical inference) in a real data analysis setting. A causal graph (Bayesian Network) is an illustration of the causal relationship among covariates, treatment, and response variable, as well as representation of assumptions. The existence of a directed edge *Y* → *X* means that *X* may have a direct causal effect on *Y*. Assume a DAG *D* = (*ν, ε*) where *ν* is a set of random variables represented by nodes (or simple by letters) in DAG *D* and *ε* is a set of edges which connect the variables. The concept of a causal graph *D* = (*ν*, *ε*) depends on the variables *ν* and edges *ε* and any inference depends on the set (*ν, ε*). Assume *P* is a joint probability distribution on *ν. D* and *P* must satisfy the basic Markov condition that every variable, *X_i_ ∈ ν*, is independent of any subset of its predecessors conditioned on the set of its direct or immediate causes (parents), [4]. The two primary underlying assumptions are that there are no latent variables and no loops in the graph. With this brief review, we now embark on a real data example.

**Figure 1:**
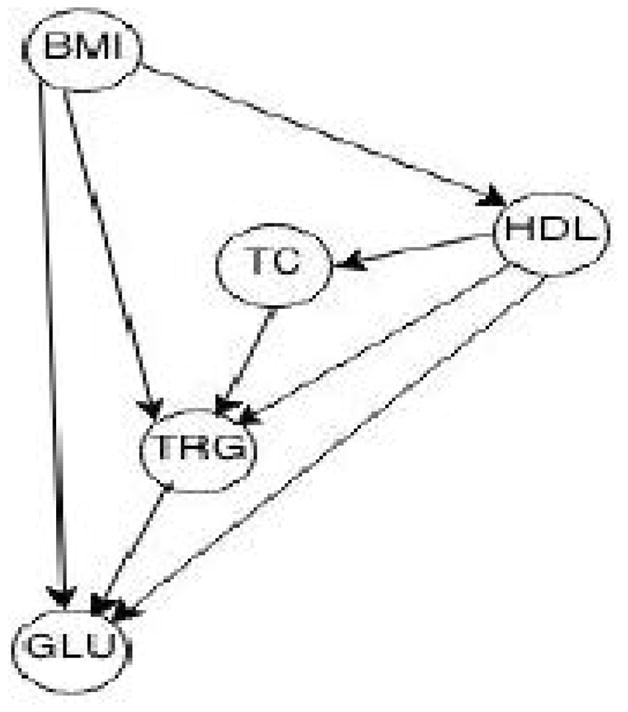
The estimated causal graph across the entire sample set.

The aim of this example application is to identify causal relationships among 5 cardiovascular disease risk factors: body mass index (BMI), glucose, triglycerides, HDL-cholesterol and total cholesterol. We apply graphical models to visualize the AM and use SEM to estimate the causal effect, since they are the most pragmatic approaches to causal analysis. The data were collected on 14,749 individuals (10,753 European-Americans and 3,996 African-Americans) from the Atherosclerosis Risk in Communities study [32]. GWAS array genotype data were also available and principal components over these genotype data were calculated and used in the analysis to account for population structure [33]. There are multiple algorithms to identify causal structure which can be categorized in constraint-based, score-based, or hybrid learnings [34–36]. Here, the Peter and Clark (PC) algorithm, which is a constraint-based algorithm, was used. The PC algorithm is available in the pcalg package implemented on CRAN [37] and was extended to consider both genotype and phenotype data. The causal graph across the entire sample set is shown in Figure 1, but the principle components from the genome are not depicted to highlight the causal relationship among the phenotypes.

**Figure 2:**
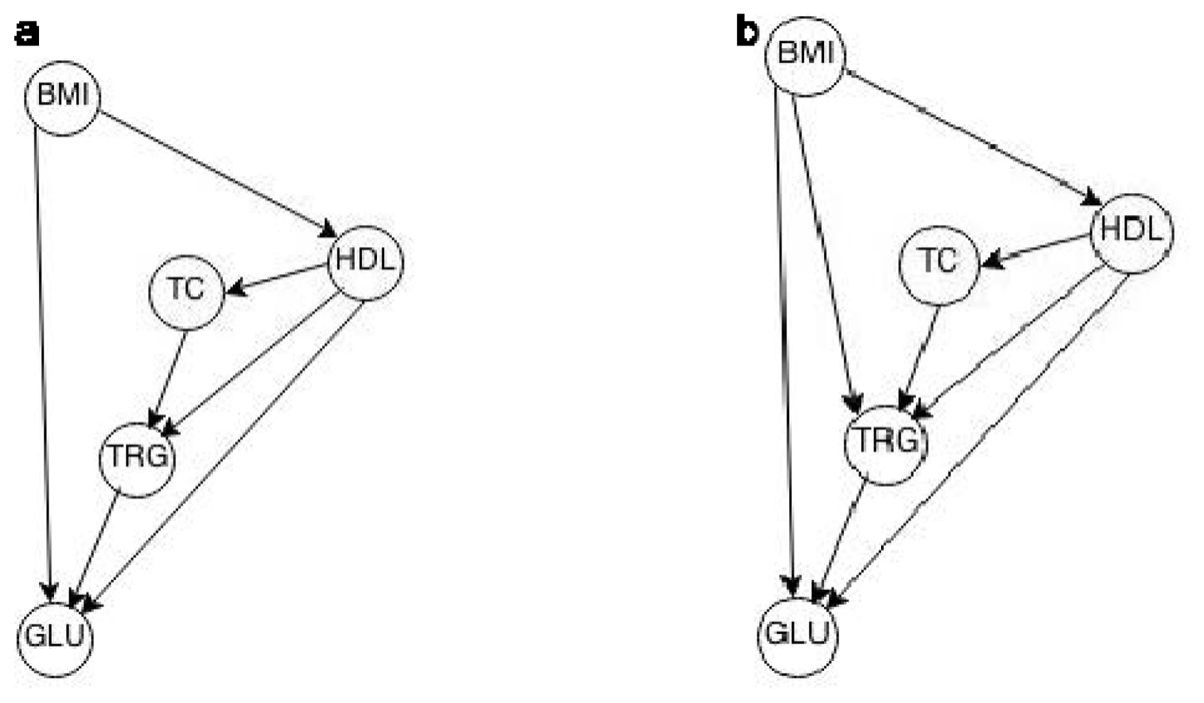
a. Estimated causal structure African-American (3856 individuals) b. Estimated causal structure European-American (10753 individuals).

The central role of BMI on plasma glucose and lipid levels is evident from the graph. BMI influences TRG levels both directly and via HDL levels. Based on this topology, the structural equation for triglyceride levels is:

(1) TRG=α·BMI+u,

where BMI is the treatment and TRG is the response variable, and there is no confounder of the AM depicted in figure 1. The estimated coefficient of BMI is *α̂* = 0.18. Because of the central role of BMI in the above graph and because BMI levels differ markedly between race groups, we hypothesize that the analyses should be repeated stratified by race. The race stratified causal graphs are shown in figure 2, a and b.

Within each race group, the topology or structures among phenotypes are similar, but not the same; there is no direct effect of BMI on triglyceride levels in the sample of African-Americans. To find the population causal effect of BMI on TRG, we must identify the causal effect within each race group:

$${\mathrm{TRG}}_{AA}=\alpha_{AA}\cdot {\mathrm{BMI}}_{AA}+u_{AA}\;\;\;{\mathrm{TRG}}_{EA}=\alpha_{EA}\cdot {\mathrm{BMI}}_{EA}+u_{EA},$$

where the subscripts AA and EA indicate African-Americans and European-Americans, respectively. There is no confounder for this causal identification based on graphical back-door and front-door criteria, [4]. The coefficients *α_AA_* = 0.09 and *α_EA_* 0.26= are interpreted causally because we assume *U* ⊥ *BMI* in each subpopulation regarding race, which means by changing BMI, the rest of the model remains intact. The population causal effect of BMI on TRG is 0.22, which is a weighted sum of race-specific causal effects, *α_EA_* · *r_EA_* + *α_AA_* · *r_AA_*, where *r_EA_* and *r_AA_* are, respectively, the proportion of European-American and African-American in the population. We can see that the population causal effect of BMI on TRG, 0.22, is absolutely higher than the effect for AA due to the high effect for EA as well as the bigger portion of EA, the number of EA is nearly three times larger than AA. If we apply the population causal effect for every individual, we would overestimate the effect for AAs and underestimate it for EAs.

Note the above computations estimate the total causal effect of BMI on TRG which comprises direct effect as well as indirect effect through mediators, here through TC and HDL. In the analysis given below, other mediators are included in the structural equation to estimate the direct effect of BMI on TRG. The structural equation for the European-Americans is

(2) $${\mathrm{TRG}}_{EA}=\alpha_{EA}\cdot {\mathrm{BMI}}_{EA}+\lambda_{EA}\cdot {\mathrm{HDL}}_{EA}+\theta_{EA}\cdot {TC}_{EA}+u_{EA},$$

and the direct effect of BMI on TRG is *α̂_EA_* = 0.11.

To better reflect the personalized effects of BMI on the other phenotypes, we next consider genotypes which influence only the variables of interest. There are 11 genotype-derived principle components which influence TRG, denoted by vector G in the equation below. By entering G into the model, we are able to account for more of the variation of TRG and increase the coefficient of determination of the model:

(3) $${\mathrm{TRG}}_{EA}=\alpha_{EA}\cdot {\mathrm{BMI}}_{EA}+\lambda_{EA}\cdot {\mathrm{HDL}}_{EA}+\theta_{EA}\cdot {TC}_{EA}+\beta_{EA}^{T}G_{EA}+u_{EA},$$

where *G_EA_* and *β_EA_* are 11×1 column matrixes. Since G comprises only variables influential on TRG and is independent of BMI, the coefficient of BMI does not change by entering G into the model. By taking into account genotype, equation 3 moves one step closer to estimate TRG for a new individual at the genotype level.

Consider the following scenario and question related to *i^th^* individual with a TRG level equal to 54 and a BMI equal to 25.75. What would be the value of *TRG_i_* if the value of *BMI_i_* was lowered by 5 while the value of other mediators were held the same? For individual causal effect by SEM see [6]. In this example, assume *i* is a European-American. Therefore, the structural equation is

(4) $${\mathrm{TRG}}_{i}=0.11\cdot {\mathrm{BMI}}_{i}-0.40\cdot {\mathrm{HDL}}_{i}+0.29\cdot {TC}_{i}+0.23,$$

where the data have been rescaled to standardized units. To answer the question, we keep the observed values of HDL and TC unchanged and set the value of BMI to calculate *TRG_i_* (*BMI_i_* – 5). The causal effect of this change on TRG level of individual *i* is

$$\text{Causal}\;\text{effect}=\mathrm{TRG}({\mathrm{BMI}}_{i}-5)-{\mathrm{TRG}}_{i}=43.78-54=-10.22$$

In other words, when individual *i* loses weight to lower *BMI_i_* by 5, the triglyceride level is predicted to decrease by 10.22.

## Conclusion

There are two barriers slowing the integration of genomic information for translational studies. The first is the small effect sizes of most genetic loci. The second is the associative nature of most genetic studies, with little information about the causative mutations. To place decision medicine in a causal framework, the causal effect must be defined precisely. Pearl defines the causal effect over the population and renders a framework to identify population causal effect, which is often operationalized graphically in a practical setting [4]. Rubin on the other hand, defines the causal effect for each individual, and applies the concept of the “potential outcome” [7]. Because of the fundamental problem in discovering and measuring an individual causal effect, we typically compare similar (i.e. not the same) treated and untreated individuals. The degree of similarity must be defined and careful consideration of covariates and potential confounders must be considered.

Personalized medicine is a theoretical ideal that has given way to decision medicine for using genomic or other biomarker information to guide treatment decisions. In a typical epidemiologic or clinical trials scenario, an interaction analysis is done (and replicated) and then subgroups or subpopulations are created based on the interaction results. In the context of graphical causal inference used here, the topologic structural relationships among the variables may be different between groups. For example, in the field of pharmacogenetics, subgroups of patients are defined by genotype or other genomic information (e.g. gene expression), and the causal effect of drug treatment is different between subgroups but assumed to be the same among individuals within a subgroup. As the rate of gene discovery and a role of genomic information in disease association increases, the frequency of causal analyses in a translational setting will also increase. The purpose of this perspective was to provide brief tutorial of causal inference and to discuss the application of specific kinds of causal inference in decision medicine.
